# Adipose-derived stem cells derived decellularized extracellular matrix enabled skin regeneration and remodeling

**DOI:** 10.3389/fbioe.2024.1347995

**Published:** 2024-04-02

**Authors:** Jin Zhang, Yang Xiang, Quyang Yang, Jiqiu Chen, Lei Liu, Jian Jin, Shihui Zhu

**Affiliations:** ^1^ Department of Burns, The First Affiliated Hospital of the Naval Medical University, Shanghai, China; ^2^ Department of Dermatology, Huashan Hospital, Fudan University, Shanghai Institute of Dermatology, Shanghai, China; ^3^ Department of Burns and Plastic Surgery, Shanghai Children’s Medical Center, School of Medicine, Shanghai Jiao Tong University, Shanghai, China

**Keywords:** adipose-derived stem cell (ADSC), extracellular matrix (ECM), decellularized extracellular matrix (dECM), carboxymethylcellulose (CMC), skin regeneration, wound healing

## Abstract

The tissues or organs derived decellularized extracellular matrix carry immunogenicity and the risk of pathogen transmission, resulting in limited therapeutic effects. The cell derived dECM cultured *in vitro* can address these potential risks, but its impact on wound remodeling is still unclear. This study aimed to explore the role of decellularized extracellular matrix (dECM) extracted from adipose derived stem cells (ADSCs) in skin regeneration.

**Methods:** ADSCs were extracted from human adipose tissue. Then we cultivated adipose-derived stem cell cells and decellularized ADSC-dECM for freeze-drying. Western blot (WB), enzyme-linked immunosorbent assay (ELISA) and mass spectrometry (MS) were conducted to analyzed the main protein components in ADSC-dECM. The cell counting assay (CCK-8) and scratch assay were used to explore the effects of different concentrations of ADSC-dECM on the proliferation and migration of human keratinocytes cells (HaCaT), human umbilical vein endothelia cells (HUVEC) and human fibroblasts (HFB), respectively. Moreover, we designed a novel ADSC-dECM-CMC patch which used carboxymethylcellulose (CMC) to load with ADSC-dECM; and we further investigated its effect on a mouse full thickness skin wound model.

**Results:** ADSC-dECM was obtained after decellularization of *in vitro* cultured human ADSCs. Western blot, ELISA and mass spectrometry results showed that ADSC-dECM contained various bioactive molecules, including collagen, elastin, laminin, and various growth factors. CCK-8 and scratch assay showed that ADSC-dECM treatment could significantly promote the proliferation and migration of HaCaT, human umbilical vein endothelia cells, and human fibroblasts, respectively. To evaluate the therapeutic effect on wound healing *in vivo*, we developed a novel ADSC-dECM-CMC patch and transplanted it into a mouse full-thickness skin wound model. And we found that ADSC-dECM-CMC patch treatment significantly accelerated the wound closure with time. Further histology and immunohistochemistry indicated that ADSC-dECM-CMC patch could promote tissue regeneration, as confirmed via enhanced angiogenesis and high cell proliferative activity.

**Conclusion:** In this study, we developed a novel ADSC-dECM-CMC patch containing multiple bioactive molecules and exhibiting good biocompatibility for skin reconstruction and regeneration. This patch provides a new approach for the use of adipose stem cells in skin tissue engineering.

## Introduction

The skin demonstrates remarkable self-repair capability through intricate interactions among cells, the extracellular matrix (ECM), bio-molecules, and cytokines (Zhu et al., 2023). Wound healing plays a crucial role in maintaining and restoring epidermal barrier integrity after skin damage and the process of wound healing exhibits intricate and dynamic changes at the molecular and cellular levels. For extensive or irreversible damage, temporary skin substitutes or scaffolds are frequently required to expedite skin repair and regeneration. Traditional treatment modalities primarily involve the use of dressings to maintain wound hydration and prevent further trauma, while their therapeutic efficacy often falls short. Consequently, the development of novel strategies for more efficacious wound healing therapies is urgently required for clinical application (Dong and Guo, 2021).

In recent years, tissue engineering strategies, particularly those based on the application of ECM, have gained increasing prominence in the field of regenerative medicine (Zieske, 2001; Lin et al., 2023). When ECM is implanted or injected into tissue, its structural proteins provide biological strength and form a three-dimensional network, thereby attracting cells during tissue regeneration (Zieske, 2001; Vriend et al., 2022). Moreover, bioactive molecules contained within ECM can convey biological signals to cells to regulate their activity (Huang et al., 2022). Decellularization, removal of antigenic components while preserving bioactive molecules, can be employed to prepare decellularized extracellular matrix (dECM)-based biomaterials (Brown et al., 2022; Liu et al., 2022; Zhang et al., 2022). dECM derived from organs or tissues preserves its native three-dimensional structure and abundant active components, but there are certain challenges in biocompatibility, immunogenicity, cell permeability, and disease transmission (Golebiowska et al., 2024). However, dECM derived from cells poses no risk of pathogens and immunogenic molecules, enabling resolution of these potential issues (Aamodt and Grainger, 2016). Therefore, considerable attention has been directed towards extraction of dECM from *in vitro* cell culture as a substitute for dECM derived from tissues or organs (Ha et al., 2020). Among these, dECM derived from stem cells holds great promise in various tissue engineering and regenerative medicine applications (Kim et al., 2020; Zhang et al., 2020; Brown et al., 2022; Zhu et al., 2023).

Adipose-derived stem cells (ADSCs) are commonly applied in the reconstruction and repair of soft tissues, playing a pivotal role in both plastic surgery and tissue engineering (Dai et al., 2016; Wankhade et al., 2016; Gadelkarim et al., 2018). Numerous studies have demonstrated that during *in vitro* cultivation, ADSCs could secret a multitude of bioactive cytokines and growth factors, including transforming growth factor-β, hepatocyte growth factor (HGF) and vascular endothelial growth factor (VEGF) (Qin et al., 2023; Wang et al., 2023; Wu et al., 2023). These mediators are instrumental in fostering cell proliferation, cellular migration, angiogenesis regulation, immune response modulation, and inflammation response regulation, assuming an indispensable role in the wound repair process (Song Y. et al., 2023; Wu et al., 2023; Kang et al., 2024). Following decellularization, these cytokines and growth factors partially remained within the dECM, which is closer to the natural ECM, thus enabling the dECM to exert a potential substantial impact in tissue regeneration.

On the other hand, it is worth mentioning that the dECM extracted from two-dimensional cell culture possesses certain limitations in its lack of intricate three-dimensional structures (Assunção et al., 2020). In order to addressing this issue, scaffold materials can be employed to load the dECM and alter its topological structure. Among these scaffold materials, carboxymethylcellulose (CMC) stands out as an excellent cellulose derivative exhibiting outstanding performance (Yang et al., 2023; Luo et al., 2024). CMC possesses several advantages such as hydrophilicity, biocompatibility, bioadhesion, biodegradability, non-toxicity, and gelation, surpassing biopolymers listed in pharmacopeias. These advantages render CMC a particularly promising hydrogel scaffold, widely utilized in tissue engineering, wound healing, aesthetic fillers, and drug delivery (Silva et al., 2023; Soullard et al., 2023). Therefore, the present study utilized CMC as a scaffold for the dECM.

This study speculated that dECM extracted from ADSCs contained multiple bioactive molecules, and we utilized CMC as a scaffold to deliver dECM for the preparation of a novel wound dressing. And this study further investigated the role of this novel dressing in wound healing and assessed the clinical application value of dECM in tissue engineering.

## Methods

### Cell isolation and cell culture

Human ADSC were isolated and cultured as previously described (Song Y. et al., 2023). Extract adipose stem cells from liposuction patients. Rinse with phosphate-buffered saline phosphate-buffered saline (PBS; Beyotime, China) and digest fat tissue with type I collagenase at 2 mg/mL for 45 min (Gibco, United States). Add fetal bovine serum (FBS; Gibco, United States) to stop the digestion. Screen through a 70 μm mesh filter then centrifuge 3,000 rpm for 5 min. Re-suspend precipitates in PBS and reseed into onto 10 cm culture dishes in Dulbecco’s modified Eagle’s medium (DMEM; Gibco, United States), supplemented with 10% fetal bovine serum (FBS; Gibco, United States) and 1% penicillin/streptomycin (Gibco, United States) at 37°C in a 5% CO_2_ humidified incubator (Thermo Fisher Scientific, United States). The medium was replenished every 2–3 days. When adherent cells reach about 80%–90% confluence, we passage them at a 1:2 or 1:3 ratio. After three passages, use ADSCs for further experiments. Inspect the cell morphology under the microscope. Lastly, identify ADSC’s surface markers CD90 (11-0909-42, Thermo Fisher Scientific, United States), CD45 (58-0459-42, Thermo Fisher Scientific, United States), CD105 (17-1057-42, Thermo Fisher Scientific, United States), CD31 (17-0319-42, Thermo Fisher Scientific, United States), CD29 (12-0299-42, Thermo Fisher Scientific, United States) and CD11b (12-0118-42, Thermo Fisher Scientific, United States) via flow cytometry.

### Preparation of ADSC-dECM

After attaining 90%–100% confluence, the medium was discarded, and the cells were gently washed twice or thrice with phosphate-buffered saline (PBS; Beyotime, China) and lysed with a solution comprising 0.25% Triton X-100 (Beyotime, China) and 10 mM NH_4_OH (Adamas, Switzerland) for 90s at 37°C (Kim et al., 2022). Upon completion of the decellularization process, super nuclease (Beyotime, China) was added and gently lysed at 37°C for 90 min. The solution was collected and frozen overnight at −80°C, and then lyophilized using freeze dryer (Scientz, China).

### DNA residue assay

Quant-iT™ PicoGreen™ dsDNA assay kit (Thermo Fisher Scientific, United States) was used. Dilute the PicoGreen reagent 200-fold with 1X TE buffer. Dilute the 2 μg/mL DNA stock solution to 50, 5, and 500 pg/mL with 1X TE buffer. Then, add 100 μL of DNA dilution and 100 μL of PicoGreen dilution to each well of the 96-well plate. Incubate for 5 min at room temperature, avoiding light. After incubation, the fluorescence of the samples was measured using an enzyme marker (excitation ∼480 nm, emission ∼520 nm). The fluorescence value of each sample was subtracted from the fluorescence value of the reagent blank to make a standard fluorescence *versus* DNA concentration curve. Prepare 1 mg/mL ADSC-dECM solution. Add 100 μL ADSC-dECM solution and 100 μL PicoGreen dilution to each well of a 96-well plate. Avoid light for 5 min of incubation at room temperature and measure the fluorescence values with the same parameters using an enzyme labeler. Subtract the blank group’s fluorescence value from each sample’s fluorescence value. The DNA concentration of the ADSC-dECM solution was determined from the DNA standard curve.

### Western blot (WB)

Samples of ADSC and ADSC-dECM were treated with RIPA lysis buffer (Beyotime, China) containing a protein inhibitor cocktail (Beyotime, China) at 4°C for 30 min. The lysates were centrifuged at 12,000 rpm for 15 min, and the supernatant was collected. The protein concentration was measured with the BCA kit protein assay (Thermo Fisher Scientific, United States) to determine the total protein concentration.

Equal amounts of protein were loaded on 4%–20% Hepes-Tris gels (EpiZyme, China) and run using electrophoresis, and then transferred to a polyvinylidene fluoride (PVDF; EpiZyme, China) membrane. The membrane was blocked with 5% nonfat blocking grade milk and then incubated overnight at 4°C with the following primary antibodies: collagen type I (COL I; 66761-1-Ig, Proteintech, China), collagen type III (COL III; 22734-1-AP, Proteintech, China), collagen type IV (COL IV; AF0510, Affinity, United States), fibronectin (66042-1-Ig, Proteintech, China), laminin (23498-1-AP, Proteintech, China) and elastin (ab307150, Abcam, United States). The appropriate secondary antibodies (1:5,000, Cell Signaling Technology, China) were chosen for 2 h of incubation at room temperature. The density of the bands was measured by ImageJ software, and GAPDH (EpiZyme, China) was selected as the internal reference.

### Enzyme-linked immunosorbent assay (ELISA)

Commercially available ELISA-KITs (Boster, China) containing hVEGF, hPDGF-BB, hPDGF-AA, hEGF, hFGF-1, and hFGF-7 were employed to measure the levels of these six growth factors. According to the manufacturer’s instructions, the OD values of the supernatant from each well were determined using an enzyme label reader (Thermo Fisher Scientific, United States) at a wavelength of 450 nm. Standard solutions of known concentration were employed to construct the standard curve. The regression equation for the standard curve was calculated and the concentrations of the samples could be determined based on their OD values by the standard curve.

### Analysis by mass spectrometry (LC-MS/MS)

The protein solution was firstly digested into a peptide mixture with protease (Ragelle et al., 2017). Then, LC-MS/MS was performed using a Q Exactive mass spectrometer coupled with an Easy nLC (Thermo Fisher Scientific, United States). The peptide sample was loaded onto the C18-reversed phase analytical column (Thermo Fisher Scientific, United States) in buffer A (0.1% formic acid in HPLC grade water), and separated with a linear gradient of buffer B (80% acetonitrile and 0.1% formic acid) with a flow rate at 300 nL/min. The linear chromatographic gradient was achieved with linear increase of buffer B percentage. After that, the peptide entered into the Q Exactive mass spectrometer (Thermo Fisher Scientific, United States). The MS analysis was set for 60 min in a positive ion mode. MS data was acquired using a data-dependent top10 method dynamically choosing the most abundant precursor ions from the full scan (350–1,800 m/z) for HCD fragmentation. The raw data obtained was then imported into Proteome Discoverer 2.2 (Thermo Fisher Scientific, United States) for protein identification, then embedded Mascot 2.6 engines was used for database searches. Protein identification was performed using reviewed database.

### Fabrication of bioactive scaffolds

Biologically active scaffolds were prepared by freeze-drying within a mold utilizing CMC powder and ADSC-dECM powder. Briefly, 2.5% of CMC was dissolved in PBS, and ADSC-dECM was added to a concentration of 0.1% w/v (1 mg/mL) with the inclusion of 2.5% CMC. Subsequently, the prepared solution comprising either CMC or ADSC-dECM-CMC was transferred into a 10cm mold, subsequently underwent freezing at −80°C for an extended period. After that, the freeze-drying process was initiated to yield the CMC patch or the CMC patch supplemented with ADSC-dECM.

### Scanning electron microscopy (SEM) analysis of the ADSC-dECM-CMC patch

The SEM (Hitachi, Japan) was used to examine the microstructure of the patch. Before observation, coat the freeze-dried patch with gold sputtering.

### Cell viability assay

To estimate the effect of ADSC-dECM on cell proliferation, human keratinocytes cells (HaCaT), human fibroblasts (HFB) and human umbilical vein endothelia cells (HUVEC) were treated with ADSC-dECM at different concentrations (0, 10, 1, 100, 10, 1 ng/mL). The culture medium without ADSC-dECM was set as the control group. Cells were counted and seeded at a density of 2,000 cells per well in a 96-well plate and cultured in a 37°C, 5% CO_2_ incubator. After 24 h, the cells adhered to the wall and then treated with ADSC-dECM. Following cultivation for 0, 6, 12, and 24 h, cell counting assay reagent (CCK-8; Solabio, Japan) was incorporated into the cell culture medium, post which it was incubated for an additional 2 h. Subsequently, optical density (OD) value of each well was measured at a wavelength of 450 nm using a microplate reader (Thermo Fisher Scientific, United States). The following formula was employed to assess the viability of cells: cell viability (%) = [(OD of the ADSC-dECM group - OD of the negative control group)/(OD of the control group - OD of the negative control group)] × 100%.

### Cell migration assay

Cell migration was estimated using a scratch assay. HaCaT, HFB and HUVEC were cultured at 1 × 10^5^ cells per well (3 wells per group) until 100% fusion was achieved. Subsequently, scratch treatment was applied using a 200 μm micropipette tip. The scratched cells were washed with PBS and cultured in serum-free medium containing different concentrations of ADSC-dECM (0, 1, 100, 10 and 1 ng/mL). The culture medium without ADSC-dECM was set as the control group. The width of the scratch was visualized with a light microscope, and the wound closure rate was measured by ImageJ software at various timepoints extending from 0, 12, 24, 36, to 48 h.

### *In vivo* full-thickness skin wound healing study

This experiment involved the procurement of 15 six-week-old male C57BL/6J mice from GemPharmatech Company. All animal experiments were conducted in strict accordance with the guidelines for experimental animals and were approved by the ethics committee. The mice were housed under controlled conditions, with a temperature of 23°C ± 2°C, humidity of 50% ± 5%, and a light-dark cycle of 12/12 h.

Prior to the experiment, mice were anesthetized with isoflurane and depilated of their dorsal hair using depilatory cream. Iodophor was used to disinfect the skin of mice, and a 10 mm diameter skin punch was applied to the back area of the mice to form a circular wound. A retractor, needle holder, and sutures were used to suture an anti-contracture ring to the wound. The CMC patches and ADSC-dECM-CMC patches were cut into circular discs with a diameter of 12 mm and sterilized under UV light with a wavelength of 254 nm for 30 min. The mice were randomly divided into three groups (n = 5 per group) including control group treated with PBS, CMC group treated with CMC patches, and ADSC-dECM-CMC group treated with ADSC-dECM-CMC patches. After patch treatment, all mice received a dressing of an oily gauze and sterile gauze to maintain humidity and prevent infection. Then, an aseptic tape dressing was applied to prevent the mice from biting the skin defect area. The treatment was repeated for three consecutive days. The detailed evaluation of the wound’s morphology captured photographically on days 7, 10, 14, and 21, and were assessed by ImageJ software. Wound closure rate (%) = (S_0_- S_t_)/S_0_*100% (S_0_: wound area on day 0; S_t_: wound area on day T).

### Histopathology and immunohistochemistry

Skin tissue specimens were collected from mice aged 7, 14, and 21 days and subjected to fixation with a 4% paraformaldehyde (PFA) solution (pH 7.40, 4°C) for 24 h, subsequently embodied in paraffin, and serially sectioned. The sections were then stained with hematoxylin-eosin (HE) and Congo red to assess the recovery phase of the skin tissue.

Immunohistochemistry was performed on the skin tissue sections. Simply put, the sections were rehydrated with an ethanol gradient and treated with 0.3% hydrogen peroxide and 0.3% Triton X-100 for 30 min, followed by three washes with PBS. The sections were then incubated with anti-CD31 (ab28364, Abcam, United States) and anti-Ki67 (ab15580, Abcam, United States) at 4°C overnight. On the subsequent day, the sections were washed three times with PBS and incubated with appropriate secondary antibody (1:500, ThermoFisher Scientific, United States) at 37°C for 30 min. And then, the sections were washed again for three times with PBS and stained with hematoxylin for 2 min. Lastly, the sections were dehydrated and analyzed under a fluorescence microscope.

### Statistical analysis

Statistical analyses were conducted using SPSS 21.0 and Graphpad Prism 9. All experiments were replicated at least three times to ensure reliable results. Single-factor analysis of variance (ANOVA), parametric measurement of Bonferroni multiple comparison *post hoc* test, nonparametric measurement of Kruskal–Wallis test, Dunn *post hoc* test, and linear regression for construction of standard curves wereapplied appropriately. Statistical significance was determined by a *p*-value less than 0.05.

## Results

### Identification of ADSC

We used flow cytometry to identify ADSCs, and the analysis results showed that ADSCs highly expressed surface markers CD90, CD105, and CD29, while low expressed CD45, CD31, and CD11b (Supplementary Figure S1).

### DNA residue

The fluorescence value of 1 mg/mL dECM solution was tested and compared with the standard curve to obtain a DNA residue of 1.57 ± 0.49 ng/mg (n = 5) for dECM, which is much lower than 50 ng/mg (Crapo et al., 2011) (Supplementary Figure S2).

### Bioactive component preserved ADSC-dECM

**FIGURE 1:**
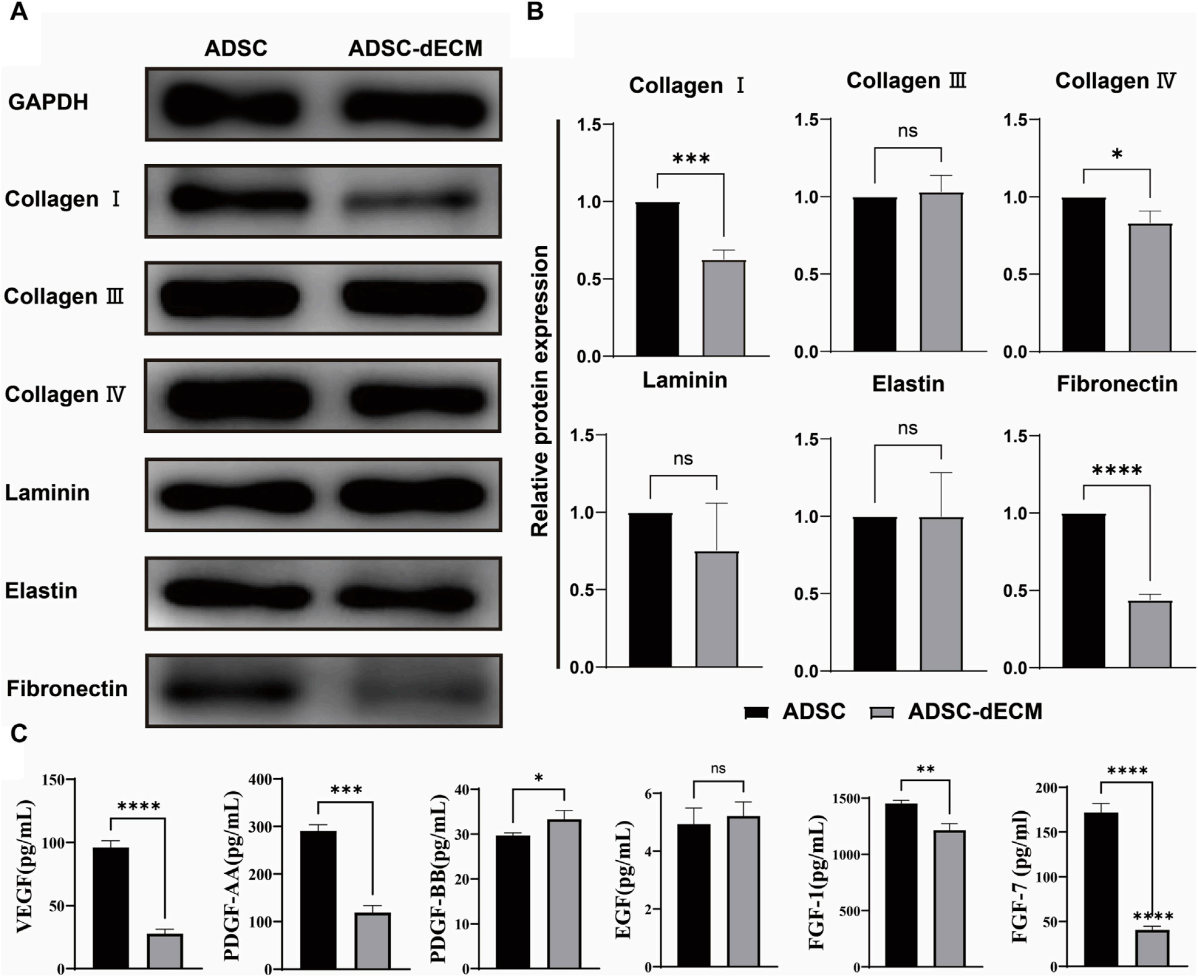
Characterization of ADSC-dECM. **(A,B)** WB analysis of ADSC-dECM proteins before and after decellularization with GAPDH as an internal control. **(C)** ELISA analysis of six growth factors of ADSC-dECM before and after decellularization. ns: *p* > 0.05, *: *p* < 0.05, **: *p* < 0.01, ***: *p* < 0.001 and ****: *p* < 0.0001 *versus* the indicated group. WB, Western blot; ELISA, Enzyme-linked immunosorbent assay; ADSC-dECM, adipose-derived stem cells derived decellularized extracellular matrix.

The isolated ADSC were employed for the preparation of ADSC-dECM. Once the cells reached 100%, the DNA and nuclei were eliminated using the optimal decellularization method documented in literature (Kim et al., 2022). And we also optimized the preservation of the ECM content. A single 10 cm culture dish of cells yielded a substantial quantity of 8 mg dECM following the extraction of ECM and subsequent freeze-drying. To verify the preservation of the active ingredients, the protein contents of cells was analyzed before and after decellularization through the application of three methods, including WB, ELISA, and MS. As a result, the WB analysis revealed negligible changes in the expression of COL Ⅲ, laminin, and elastin, while the content of COL Ⅰ, COL Ⅳ and fibronectin decreased by 40%, 20%, and 55%, respectively, following decellularization (Figures 1A, B). The ELISA assay demonstrated an increase in the content of PDGF-BB and a corresponding decrease in VEGF, PDGF-AA, FGF-1 and FGF-7 after decellularization (Figure 1C). LC-MS/MS was further used to evaluate the composition and quantitative analysis and showed both ADSC and ADSC-dECM encompassed a multitude of collagen proteins, which indicating that the collagen components after decellularization were well preserved (Table 1).

**TABLE 1:** Collagen proteins identified in ADSC and ADSC-dECM.

Accession	Gene	ADSC (ng/mL)	ADSC-dECM (ng/mL)
P02452	COL1A1	6,596.16 ± 838.24	6,296.34 ± 918.12
A0A384MDU2	COL1A2	3,173.16 ± 191.04	3,049.78 ± 442.38
P02458	COL2A1	18.16 ± 1.92	16.86 ± 1.50
P02461	COL3A1	1717.16 ± 236.64	1663.77 ± 272.28
P08572	COL4A2	3.16 ± 0.56	3.47 ± 0.26
B2RTX6	COL4A6	64.16 ± 3.97	60.31 ± 12.72
P02462	COL4A1	42.16 ± 3.64	42.78 ± 0.80
H7BY82	COL5A1	16.16 ± 1.81	20.33 ± 2.97
B2ZZ86	COL5A1	609.16 ± 90.93	572.03 ± 108.52
P05997	COL5A2	234.16 ± 5.04	217.47 ± 23.62
P12111	COL6A3	2,611.16 ± 513.29	2,562.49 ± 725.72
P12109	COL6A1	1268.16 ± 151.20	1190.42 ± 210.14
P12110	COL6A2	635.16 ± 111.71	618.77 ± 129.58
A6NMZ7	COL6A6	19.16 ± 4.90	20.75 ± 1.47
D3DT71	COL11A1	49.16 ± 3.45	45.83 ± 9.55
Q99715	COL12A1	1495.16 ± 68.81	1586.40 ± 252.90
H0Y5N9	COL12A1	11.16 ± 2.52	11.82 ± 0.51
Q05707	COL14A1	35.16 ± 4.87	36.16 ± 8.69
A0A087 × 0K0	COL15A1	50.16 ± 9.74	47.96 ± 13.36
Q07092	COL16A1	25.16 ± 1.35	24.64 ± 3.90
D3DSM4	COL18A1	12.16 ± 0.44	12.45 ± 1.90
Q86Y22	COL23A1	1.16 ± 0.04	1.36 ± 0.33
A0A024R462	FN1	4,879.16 ± 2,903.75	4,264.52 ± 1830.78
P11047	LAMC1	376.16 ± 17.96	458.14 ± 123.43
P55268	LAMB2	107.16 ± 6.40	104.45 ± 12.83
Q53EP0	FNDC3B	80.16 ± 3.14	82.01 ± 3.92
P07942	LAMB1	57.16 ± 5.02	57.35 ± 2.42
H0UI49	LAMA4	13.16 ± 1.36	13.36 ± 2.69
G5E9X3	FNDC3A	17.16 ± 1.68	17.78 ± 1.52
Q4ZHG4	FNDC1	0.16 ± 0.11	0.80 ± 0.09
P09619	PDGFRB	239.16 ± 14.07	242.20 ± 13.18
A0A1G4P1I6	NDEL1-PDGFRB	7.16 ± 0.44	7.20 ± 1.14
A0A0S2Z3V1	EFEMP1	31.16 ± 0.78	30.03 ± 2.31
O43854	EDIL3	21.16 ± 2.41	22.17 ± 1.03
P16234	PDGFRA	4.16 ± 1.00	4.49 ± 1.07
A0A0A0MQV6	FGF2	24.16 ± 4.26	27.45 ± 4.63
V5YQU3	FGFR2-BICC1	6.16 ± 1.56	7.24 ± 0.80

Accession, Protein database number; Gene Name, Protein Gene Name; Description, Protein Function Description; ADSC, adipose-derived stem cells; ADSC-dECM, adipose-derived stem cells derived decellularized extracellular matrix.

### Treatment with ADSC-dECM promotes cell proliferation and migration *in vitro*

**FIGURE 2:**
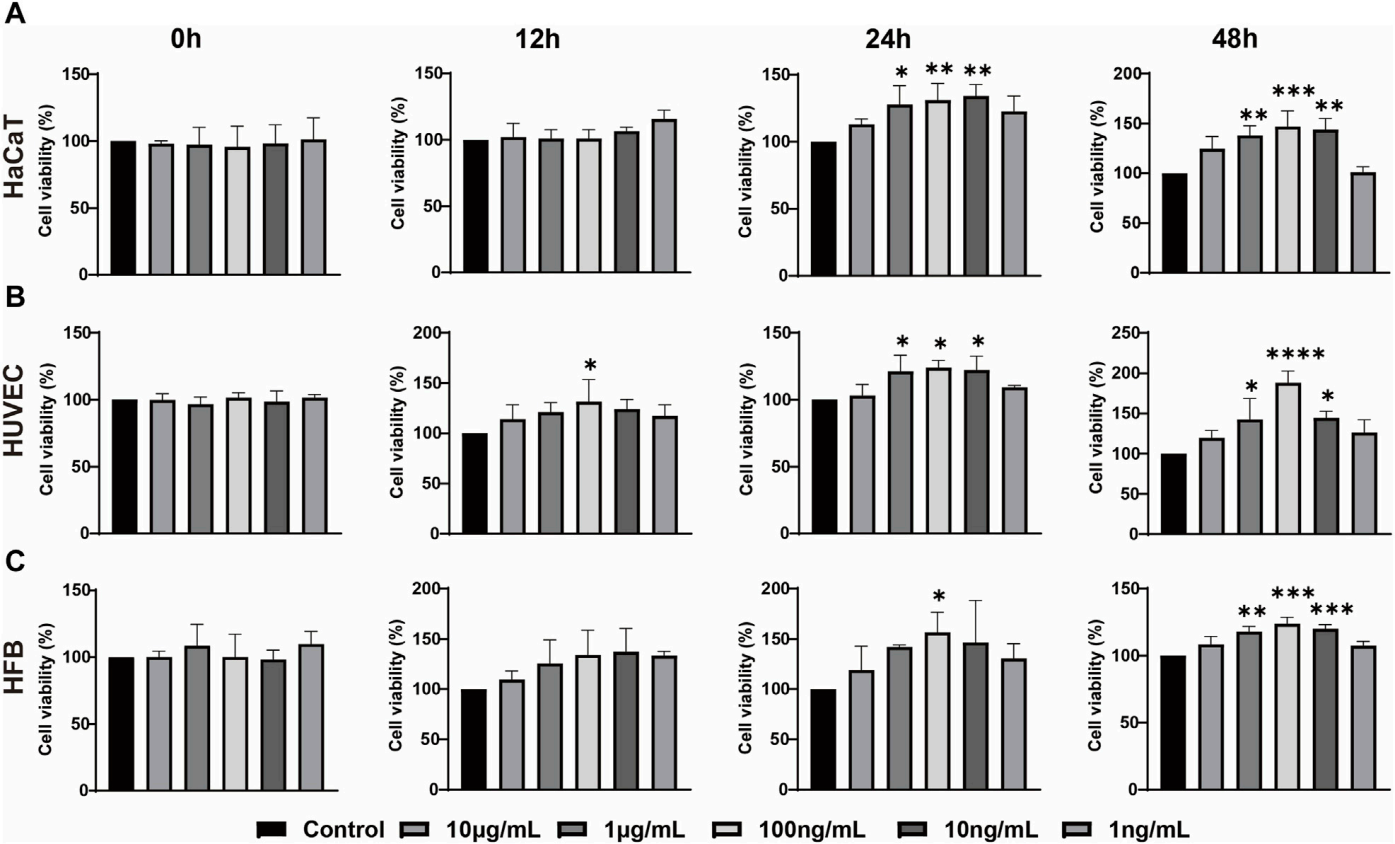
Cell counting assay for determining the proliferation of **(A)** HaCaT, **(B)** HUVEC and **(C)** HFB with different concentrations of ADSC-dECM treatments. ns: *p* > 0.05, *: *p* < 0.05, **: *p* < 0.01, ***: *p* < 0.001 and ****: *p* < 0.0001 *versus* the control group. HaCaT, human keratinocytes cells; HUVEC, human umbilical vein endothelial cells; HFB, human fibroblasts. ADSC-dECM, adipose-derived stem cells derived decellularized extracellular matrix.

**FIGURE 3:**
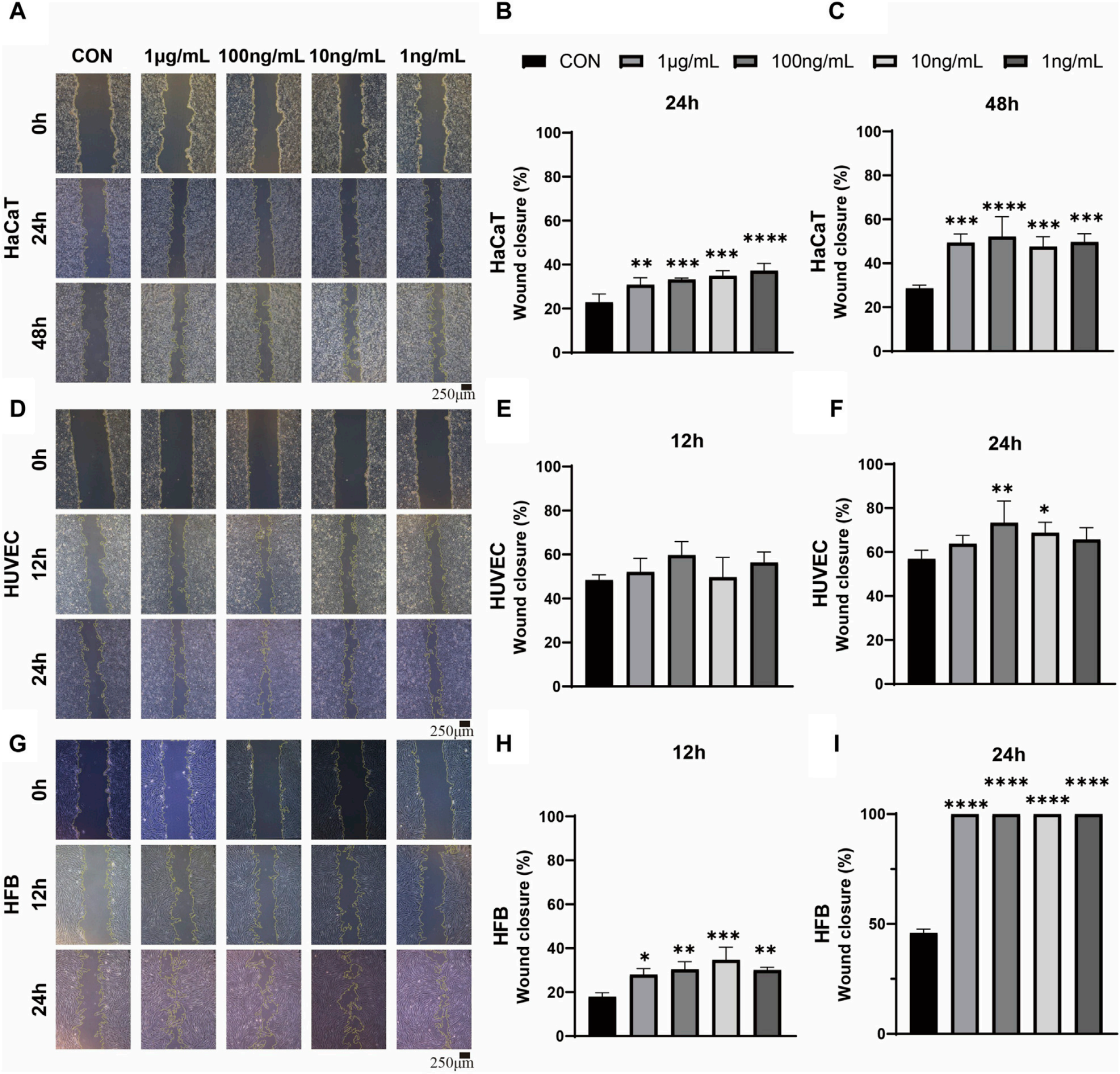
Wound scratch assay images and quantitative analysis for evaluation of the migration in monolayer HaCaT, HUVEC and HFB. **(A,D,G)** Images of HaCaT migration at 24 and 48 h, and HUVEC and HFB migration at 12 and 24 h. Scale bar = 250 μm. **(B,C,E,F,H,I)** Statistical analysis of HaCaT, HUVEC and HFB migration treated with different concentrations of ADSC-dECM. ns: *p* > 0.05, *: *p* < 0.05, **: *p* < 0.01, ***: *p* < 0.001 and ****: *p* < 0.0001 *versus* the control group. HaCaT, human keratinocytes cells; HUVEC, human umbilical vein endothelial cells; HFB, human fibroblasts. ADSC-dECM, adipose-derived stem cells derived decellularized extracellular matrix.

In order to investigate the impact of ADSC-dECM on the behaviors of HaCaT, HUVEC and HFB, after coculture with ADSC-dECM with varying concentrations, cell proliferation and migration were evaluated. The CCK-8 assay was employed to detect the proliferation of these cells at measurement time points. The results showed ADSC-dECM could significantly promote cell proliferative rates in HaCaT, HUVEC and HFB; and the effect of promoting cell proliferation became more significant with increasing ADSC-dECM concentration from 1 to 100 ng/mL (Figure 2). However, with the further increase of concentration in these cells, the effect of promoting proliferation of ADSC-dECM was weakened (Figure 2). In addition, the cell migration results confirmed that ADSC-dECM treatment could significantly promote the migration of both HaCaT and HFB; whereas its effect on HUVEC exhibited a relative attenuation (Figure 3). These results demonstrated that ADSC-dECM treatment could significantly enhance the proliferation and migration of HaCaT, HUVEC and HFB.

### *In Vivo* wound healing evaluation of the full-thickness skin wound model

**FIGURE 4:**
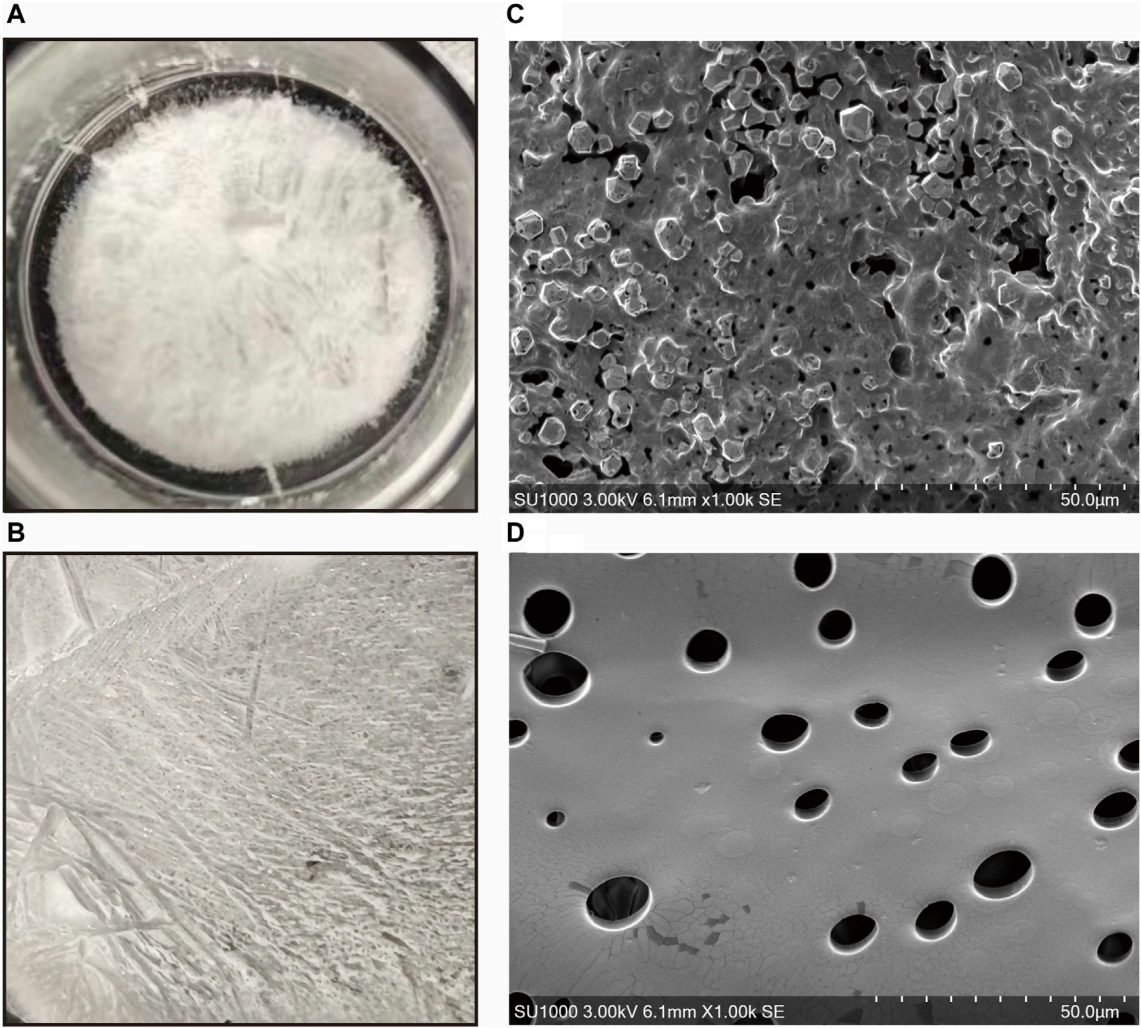
Gross appearance of the **(A)** ADSC-dECM and **(B)** ADSC-dECM-carboxymethylcellulose (ADSC-dECM- CMC) patch. Scanning electron microscopy images of **(C)** ADSC-dECM and **(D)** ADSC-dECM-CMC patch. Scale bar = 50 μm ADSC-dECM: adipose-derived stem cells derived decellularized extracellular matrix; CMC: carboxymethylcellulose.

**FIGURE 5:**
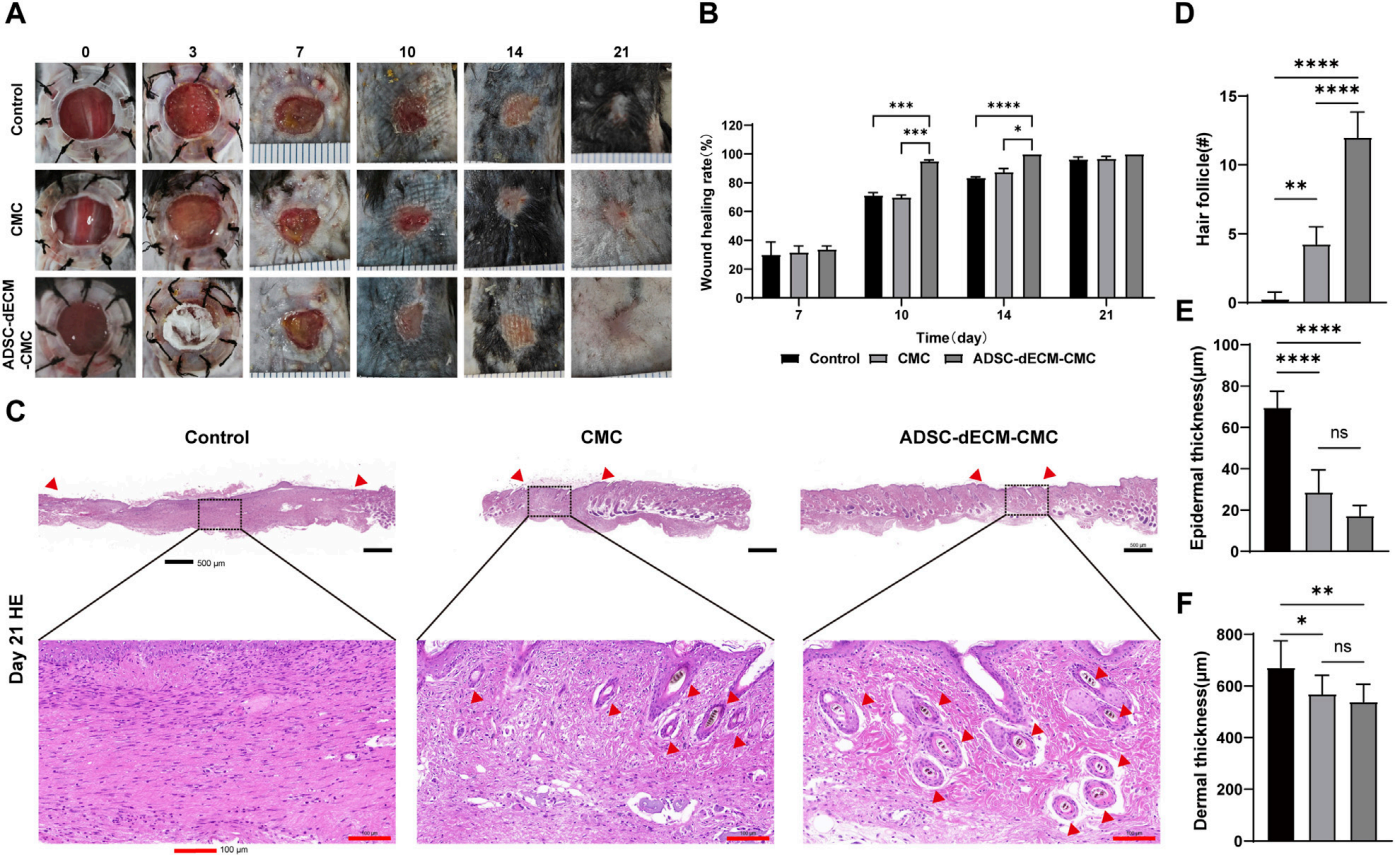
ADSC-dECM-CMC patch promoted wound healing *in vivo*. **(A)** Wound area monitored at the indicated days post-wounding and **(B)** quantitative analysis in ADSC-dECM-CMC patch group, CMC patch and the control group. **(C)** H&E staining images of wound samples at days 21. Scale bar = 500 μm and scale bar = 100 μm. Quantitative analysis of **(D)** the hair follicle count, **(E)** epidermal thickness and **(F)** dermal thickness. ns: *p* > 0.05, *: *p* < 0.05, **: *p* < 0.01, ***: *p* < 0.001 and ****: *p* < 0.0001 *versus* the indicated group. ADSC-dECM: adipose-derived stem cells derived decellularized extracellular matrix; CMC: carboxymethylcellulose.

The gross observations of ADSC-dECM powder and ADSC-dECM-CMC patch after undergoing lyophilization were shown in Figure 4. Microporous structures could be seen in SEM. The wound healing capacity of the ADSC-dECM was examined using the full-thickness skin wound model. In the normal wound model, 10-mm full-thickness skin wounds were produced on the dorsum of C57BL/6 mice, and these mice were randomly grouped and treated with the CMC patch, ADSC-dECM-CMC patch, or PBS. Images of the wound were obtained at days 0, 1, 3, 5, 7, 10, 14 and 21 (Figure 5A). After 10 days of treatment, the wound area of each group decreased, with the ADSC-dECM-CMC patch group having the highest wound closure rate and a smooth and glossy wound surface, indicating complete epithelialization and wound healing. However, the control and CMC patch groups exhibited small amounts of exudate and bleeding, with their healing rates being notably lower than that of the ADSC-dECM CMC patch group (*p* < 0.05). On day 14, both control and experimental groups achieved almost complete epithelialization.

The HE staining was further performed on the sample tissue on day 21, at which point the proliferation in the skin stopped and remodeling was active, with the aim of exploring the long-term effect of ADSC-dECM on wound healing (Figure 5C). The HE staining results showed that the skin of the control group was composed of immature skin tissue, forming scar tissue with only a small amount of skin appendages. However, the ADSC-dECM-CMC patch group showed more hair follicles on the wound skin, notably surpassing both the control and CMC groups. Furthermore, there was no notable disparity of epidermal and dermal thickness in the control and CMC groups; however, both were thinner than that of the control group. These findings collectively implied that ADSC-dECM-derived scaffolds exhibited regenerative potency and could elicit angiogenesis and wound retraction. Meanwhile, a reduction in the epidermal tissue thickness and an augmentation in hair follicles may facilitate wound remodeling.

**FIGURE 6:**
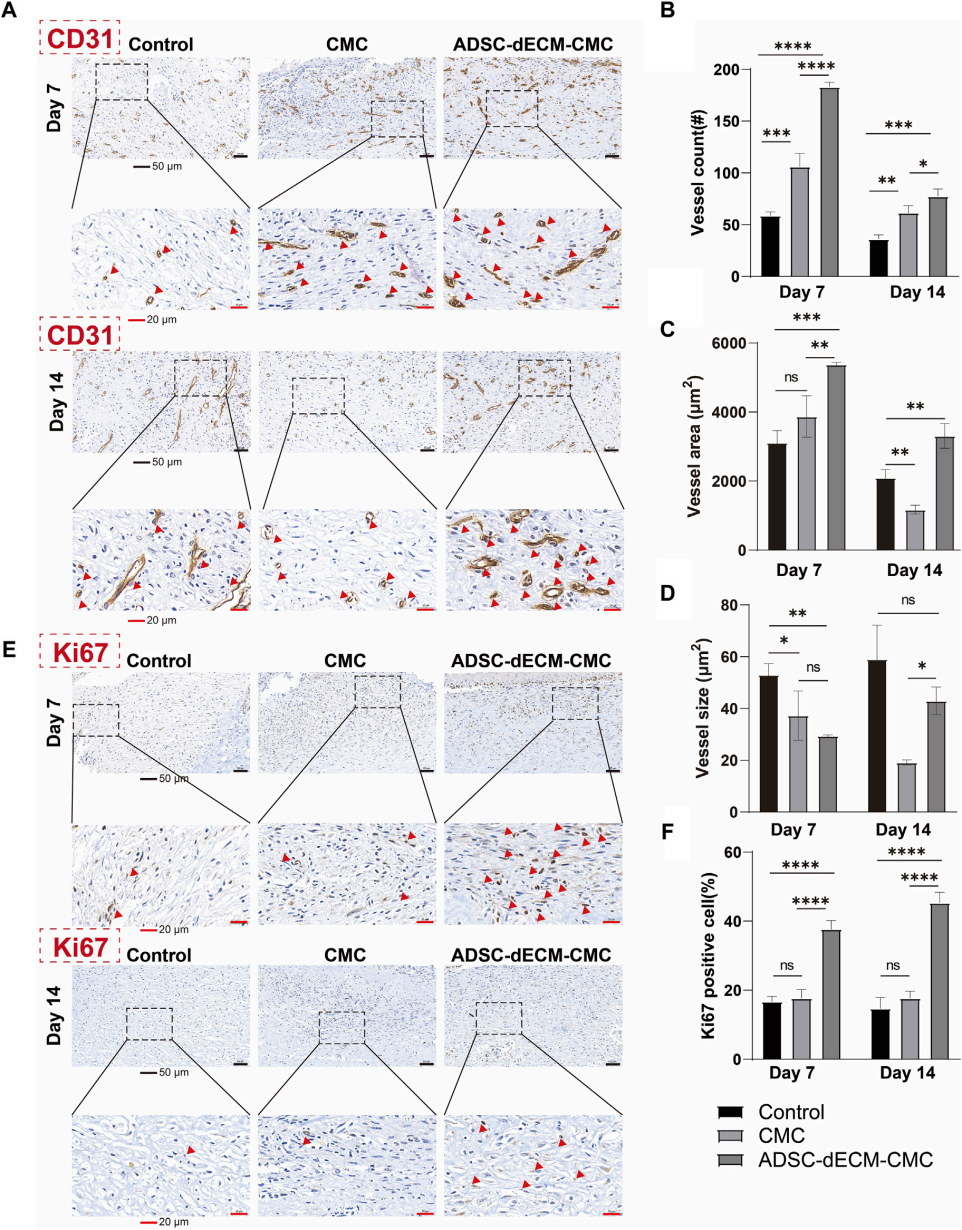
Images of immunohistochemical staining of **(A)** CD31 and **(E)** Ki67 at day 7 and day 14. Quantification results of **(B–D)** CD31 and **(F)** Ki67 at day 7 and day 14. Scale bar = 50 μm and scale bar = 20 μm #: number. ns: *p* > 0.05, *: *p* < 0.05, **: *p* < 0.01, ***: *p* < 0.001 and ****: *p* < 0.0001 *versus* the indicated group. ADSC-dECM, adipose-derived stem cells derived decellularized extracellular matrix; CMC, carboxymethylcellulose. Red arrows represent IHC CD31 positive vessles **(A)** or Ki67 positive cells **(E)**, respectively.

Angiogenesis represents a pivotal component of the wound healing process. Vessels are capable of delivering progenitor cells, oxygen, and nutrients, thereby fostering cellular proliferation and supporting matrix restoration. CD31 is a specific marker for endothelial cells and can be used to detect neovascularization. As demonstrated in Figure 6A, the ADSC-dECM-CMC patch group exhibited a substantial augmentation in vascular volume detected via the CD31-positive vessel area. The ADSC-dECM-CMC group demonstrated a significant enhancement in vascularization at the 7- and 14-day marks relative to the control and CMC groups, as indicated by an increased vessel area and count. Notably, after 14 days, there was an observable augmentation in vessel size within the ADSC-dECM-CMC and Control groups, suggestive of progressive vessel maturation (Figures 6B–D). IHC staining with cell proliferation marker Ki67 was carried out on days 7 and 14 to further evaluate the proliferation ability. As shown in Figure 6F, we found that in comparison to other groups, the ADSC-dECM-CMC patch group exhibited a notable enhancement in Ki67 expression, while the control group had relatively fewer Ki67-positive cells. These data confirmed that ADSC-dECM-CMC patch could effectively promote cell proliferation and angiogenesis.

## Discussion

ECM represents a complex mixture composed of macromolecules that not only provides structural support to cells but also regulates their physiological activities (Guan et al., 2022). The application of biomaterials based on ECM in the field of regenerative medicine is rapidly expanding (Aamodt and Grainger, 2016). Stem cells exhibit self-renewal and differentiation capabilities, ensuring continuous acquisition of dECM derived from these cells during *in vitro* culture and expansion (Assunção et al., 2020). dECM can provide specific differentiation guidance for transplanted cells, and the ECM secreted by cells derived from different tissue sources possesses specific differentiation guidance capabilities (Hussey et al., 2018; Golebiowska et al., 2024). Although some studies have demonstrated that ADSCs exhibited exceptional effects in wound repair, researches on ADSC-dECM remain limited (Nyambat et al., 2018). Therefore, in this study, we developed a novel type of biodegradable film, which physically combined ADSC-dECM with CMC, thus developing a continuous accessible, easily stored, and profoundly biologic dressing, which can be utilized in the field of skin tissue engineering and regeneration. And we further investigated the role of this novel dressing in wound healing and found it could effectively promote tissue healing.

The dECM material prepared in this experiment exhibited comprehensive decellularization efficacy without cell residue, while the major extracellular matrix component collagen proteins were well preserved. Our research showed that dECM, rather than scaffolds, could effectively promote wound healing, which is consistent with previous studies (Nyambat et al., 2018; Kim et al., 2022). The dECM contains a variety of collagen proteins and growth factors, such as collagen Ⅰ, collagen Ⅲ, collagen Ⅳ, fibronectin, elastin, laminin, EGF, VEGF and FGF. Among these, collagen protein serves as an important biopolymer with excellent biocompatibility, non-toxicity, low antigenicity, and favorable biodegradability, and it has been widely employed in the cosmetic and biomaterial fields (Sorushanova et al., 2019; Song J-H. et al., 2023). In the wound healing process, collagen protein and collagen-derived fragments play a pivotal regulatory role in numerous cellular functions, including cell shape maintenance, cell differentiation, migration, and synthesis of numerous proteins (Sorushanova et al., 2019). Collagen protein can stimulate cellular activity including inducing directed migration of cells to the wound site and promoting deposition of new collagen matrix (David, 2023). Also, collagen protein exhibits effective absorption of wound exudates, binds and protects the growth factors carried by wound exudates, thereby promoting wound healing (Gajbhiye and Wairkar, 2022). Furthermore, collagen protein can facilitate cell transportation and formation of new tissue at the wound site (Liden et al., 2016; Gajbhiye and Wairkar, 2022). Collagen proteins I and III are considered to regulate the molecular structure and mechanical properties of numerous tissues (Liden et al., 2016). And our results also confirmed abundant collagen protein in our ADSC-dECM which might contribute to its pro-regenerative function. Additionally, fibronectin and laminin are important ECM protein components involved in cell adhesion and proliferation, which may enhance roughness and facilitate increased cell adhesion and proliferation (Patten and Wang, 2021; Dörschmann et al., 2022). And WB analysis also demonstrated increased fibronectin and laminin expressions in our ADSC-dECM. Furthermore, previous researched have revealed that hydrogels laden with growth factors induced host tissue regeneration (Van Velthoven et al., 2023). These bioactive growth factors encompassed VEGF, HGF, PDGF, and FGF, among others. EGF expedites wound regeneration in various tissues, including skin, through promotion of endothelial cell and fibroblast proliferation (Jeong et al., 2020). VEGF serves as a multi-functional growth factor, manifesting the capacity to induce angiogenesis (Yen et al., 2022). Specifically, VEGF-α is the most potent angiogenic factor known to date (Vannella and Wynn, 2017). TGF-β1 stands as the most potent pro-fibrotic factor, exerting a pivotal role throughout the entire process of wound healing (Eid et al., 2022). It not only promotes rapid fibroblast proliferation, but also elicits secretion of extracellular matrix components such as collagen and hyaluronic acid by various cells, thereby facilitating granulation tissue formation, myofibroblast transformation, and re-epithelialization. FGF enhances cell proliferation, migration, and differentiation, with vast tissue engineering application prospects (Van Velthoven et al., 2023). In our study, following cell dissociation, these growth factors persisted in the ADSC-dECM confirmed via ELISA analysis, which might contribute to wound repair and regeneration.

The skin is one of the most intricate organs composed of multiple cells. Our results demonstrated that ADSC-dECM significantly stimulated HaCaT, HFB and HUVEC proliferation and migration. ADSC-dECM-CMC patches facilitated granulation tissue formation and collagen deposition, and promoted cellular proliferation and angiogenesis via immunohistochemical detection of antigens pertinent to cell proliferation (Ki67) and angiogenesis (CD31). We believed that the ECM prepared by ADSC decellularization served as a reservoir of growth factors and collagen, effectively promoting wound healing and skin regeneration. Importantly, it should be emphasized that subsequent to the completion of wound epithelialization, a diminution in neovascularization is observed, attributable to the involution of granulation tissue. This process is characterized by an enlargement of vessel size, reflective of the maturation and functionalization of the vessels. Consequently, the angiogenic potential of the decellularized matrix is most effectively harnessed before the completion of wound epithelialization to enhance the proliferation of granulation tissue and expedite the wound healing process.

Compared with dECM derived from other cell sources, dECM derived from stem cells can be continuously obtained due to the stem cell’s self-renewal capacity. Prior studies have demonstrated the remarkable ability of ADSCs in angiogenesis, epithelialization, and influencing macrophage and endothelial progenitor migration and proliferation during healing processes, and these cells have been extensively investigated for wound repair (Qiao et al., 2019; Li et al., 2022). In addition, ADSCs are abundant, relatively easily extracted and isolated, thereby avoiding ethical issues associated with their use (Li et al., 2022). Recent *in vitro* and *in vivo* animal model experiments have also demonstrated that ADSCs exhibited superior efficacy in relieving wound inflammation, promoting granulation tissue growth and collagen deposition compared to bone marrow stem cells (Fernandes et al., 2018). Through the decellularization process, dECM lacks cellular components, thereby avoiding the risks associated with stem cell usage. And our study also demonstrated the remarkable efficacy of ADSC-dECM in wound repair *in vivo*, suggesting that ADSC-dECM possesses excellent prospects for application in the field of wound repair. Hence, the ADSC-dECM used in this study was easily extracted, consistently available, risk-free of stem cells, and effective in wound healing.

There are several limitations in our study. Firstly, the cell viability of ADSC varies due to age and gender; and this study utilized a single cell line to extract dECM for semi-quantitative evaluation, without comparative analysis of cells across different age stages, introducing a potential bias. Secondly, as dECM constitutes a complex mixture of bioactive components, potential mechanism of its function in wound healing has not been thoroughly explored. Thirdly, we only examined the influence of ADSC-dECM on three types of skin cells, neglecting other cells within the skin. Lastly, although this study demonstrated remarkable efficacy of ADSC-dECM in murine wound models, further evaluation of its application value in a diverse range of wound models is necessary.

## Conclusion

In this study, we developed a novel ADSC-dECM-CMC patch for wound healing. *In vitro*, ADSC-dECM-CMC could enhance adhesion and proliferation of HACAT, HUVEC and HFB. And *in vivo* experiments verified that the ADSC-dECM-CMC patch exhibited superior wound healing capabilities within a mouse skin wound model via promoting angiogenesis, and rebuilding skin appendages. These results showed ADSC-dECM-CMC represented an ideal biomaterial scaffold for wound repair, displaying broad application prospects in tissue regeneration and wound healing.

## Data Availability

The original contributions presented in the study are included in the article/Supplementary Material, further inquiries can be directed to the corresponding author.

## Supplementary material

SUPPLEMENTARY FIGURE S1 Identification of ADSC by flow cytometry (including CD90, CD105, CD31, CD45 and CD11b).

SUPPLEMENTARY FIGURE S2 Quantification of residual DNA. DNA was quantified by PicoGreen assay.
