# Supplementary figures and images for: Adipose-derived stem cells derived decellularized extracellular matrix enabled skin regeneration and remodeling
Source: Front Bioeng Biotechnol. 2024 Apr 2;12:1347995. doi: 10.3389/fbioe.2024.1347995 (PMC11019001; doi:10.3389/fbioe.2024.1347995)

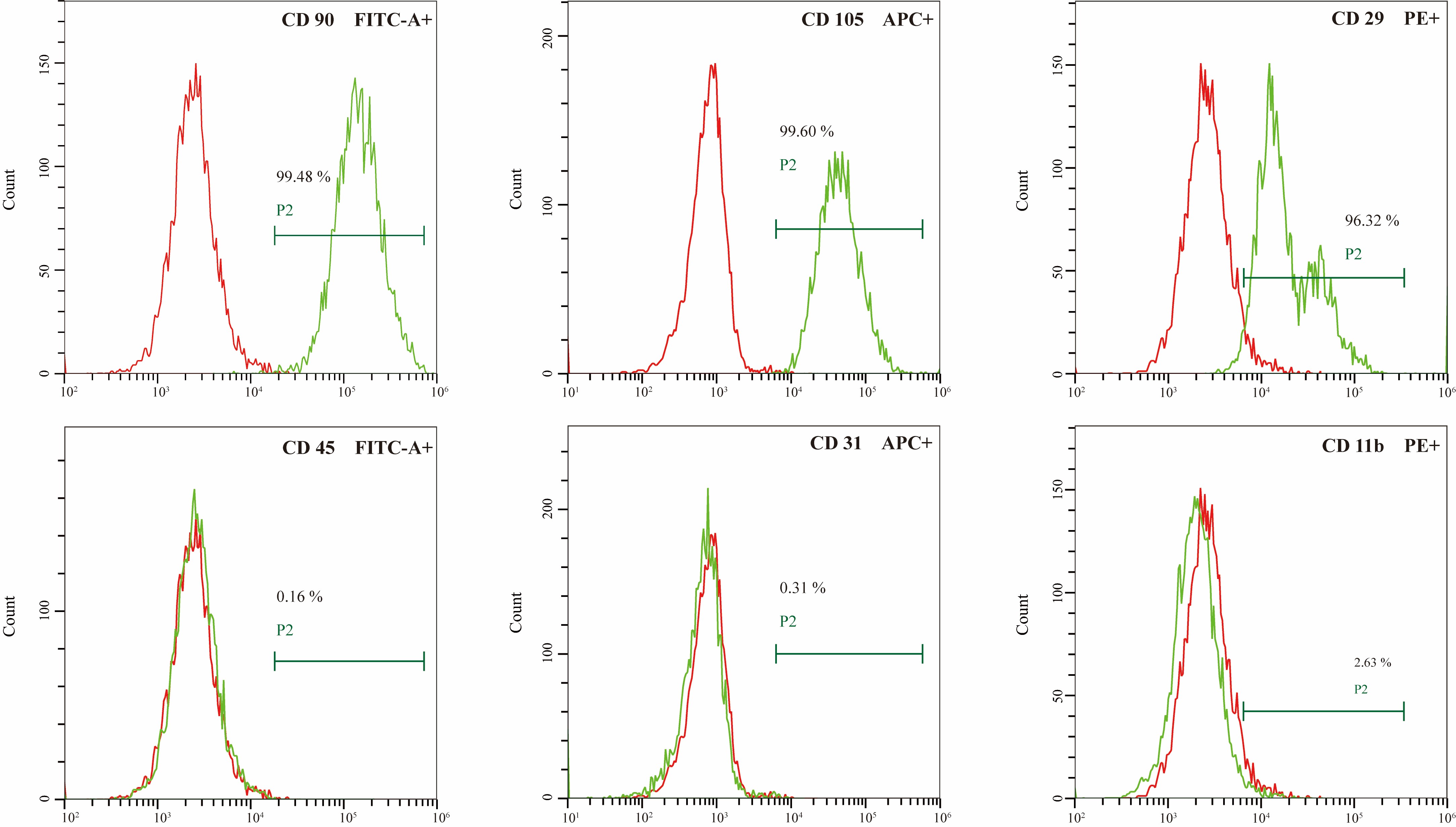

Supplement: Supplementary file 1 [file Image1.JPEG]

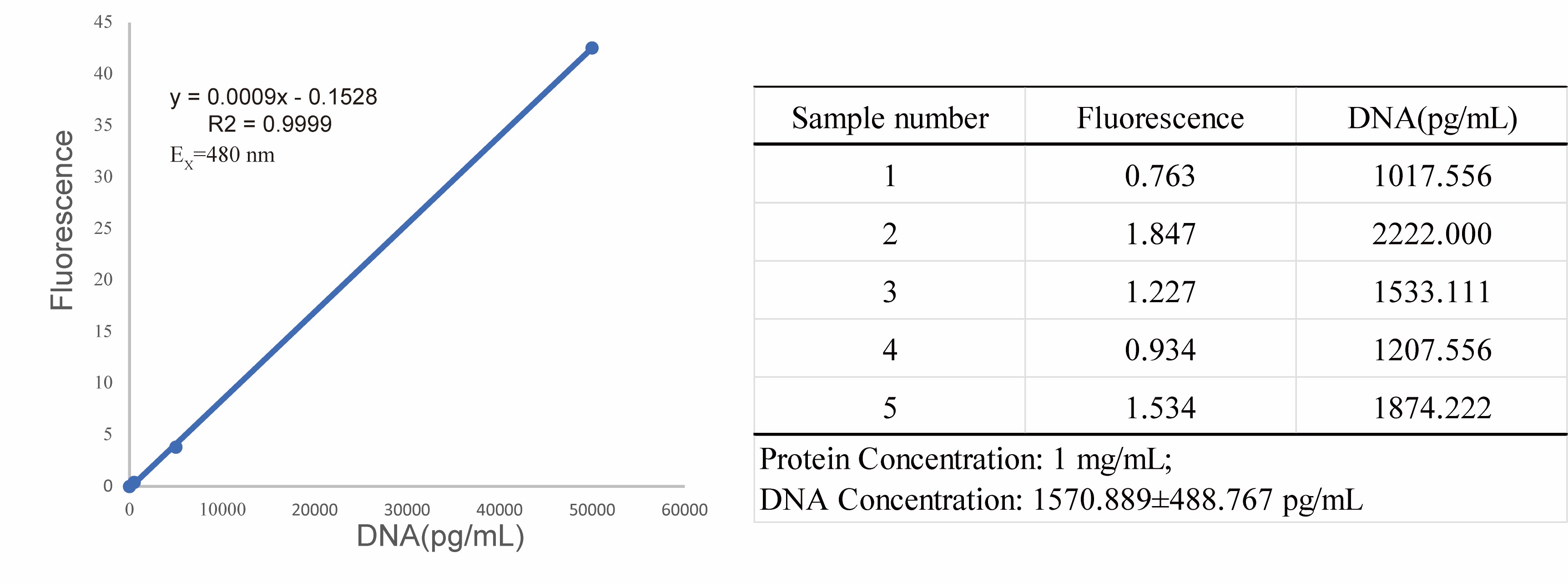

Supplement: Supplementary file 2 [file Image2.JPEG]
